# Sweat chloride assay by inductively coupled plasma mass spectrometry: a confirmation test for cystic fibrosis diagnosis

**DOI:** 10.1007/s00216-020-02821-3

**Published:** 2020-07-21

**Authors:** Antonella Marvelli, Beatrice Campi, Gianfranco Mergni, Maria Elisa Di Cicco, Paola Turini, Paolo Scardina, Riccardo Zucchi, Massimo Pifferi, Giovanni Taccetti, Aldo Paolicchi, Giancarlo la Marca, Alessandro Saba

**Affiliations:** 1 Department of Surgical, Medical and Molecular Pathology and Critical Care Medicine, University of Pisa, Pisa, Italy; 2 C.N.R. Institute of Clinical Physiology, Pisa, Italy; 3 Cystic Fibrosis Laboratory, Meyer Children’s University Hospital, Florence, Italy; 4 Pediatric Division, Department of Obstetrics, Gynecology and Pediatrics, St. Chiara University Hospital, Pisa, Italy; 5 Laboratory of Clinical Pathology, Department of Laboratory Medicine, St. Chiara University Hospital, Pisa, Italy; 6 Agilent Technologies Italia S.p.A. Cernusco sul Naviglio, Milan, Italy; 7 Department of Translational Research and of New Surgical and Medical Technologies, University of Pisa, Pisa, Italy; 8 Department of Experimental and Clinical Biomedical Sciences, University of Florence, Florence, Italy; 9 Newborn Screening, Clinical Chemistry and Pharmacology Laboratory, Meyer Children’s Hospital, Florence, Italy

**Keywords:** Cystic fibrosis, Inductively coupled plasma, Mass spectrometry, Chloride assay

## Abstract

The current guidelines for sweat chloride analysis identify the procedures for sweat collection, but not for chloride assay, which is usually performed by methods originally not aiming at the low concentrations of chloride found in sweat. To overcome this limitation, we set up, characterized, and adopted an original inductively coupled plasma mass spectrometry (ICP-MS) method for sweat chloride determination, which was designed for its easy use in a clinical laboratory. The method was linear in the range 8.5E−3 to 272.0E−3 mM, precision exhibited a relative standard deviation < 6%, and accuracy was in the range 99.7–103.8%. Limit of blank, limit of detection, and limit of quantitation were 2.1 mM, 3.2 mM, and 7.0 mM, respectively, which correspond to real concentrations injected into the mass spectrometer of 3.9E−3 mM for LOD and 8.5E−3 mM for LOQ. At first, the method was tested on 50 healthy volunteers who exhibited a mean chloride concentration of 15.7 mM (25–75th percentile 10.1–19.3 mM, range 2.8–37.4 mM); then, it was used to investigate two patients with suspected cystic fibrosis, who exhibited sweat chloride values of 65.6 mM and 81.2 mM, respectively. Moreover, the method was cross-validated by assaying 50 samples with chloride concentration values in the range 10–131 mM, by both ICP-MS and coulometric titration, which is the technology officially used in Tuscany for cystic fibrosis newborn screening. The reference analytical performances and the relatively low cost of ICP-MS, accompanied by the advantageous cost of a single sweat chloride assay, make this technology the best candidate to provide a top reference method for the quantification of chloride in sweat. The method that we propose was optimized and validated for sweat samples ≥ 75 mg, which is the minimum amount requested by the international protocols. However, the method sensitivity and, in addition, the possibility to reduce the sample dilution factor, make possible the quantification of chloride even in samples weighting < 75 mg that are discarded according to the current guidelines.

**Figure:**
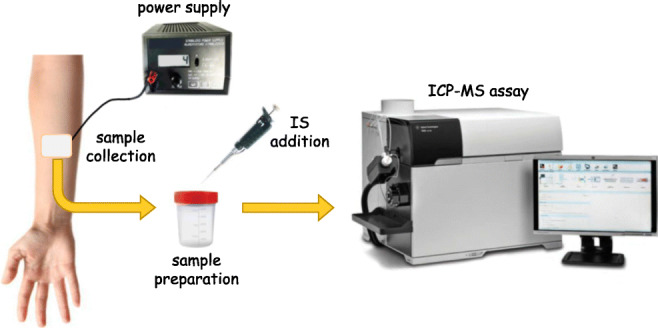
Graphical abstract

Graphical abstract

## Introduction

Cystic fibrosis (CF), an autosomal recessive disorder occurring in 1/2000–3000 livebirths among the Caucasian populations [1], is caused by mutations of the gene encoding for the Cystic Fibrosis Transmembrane Conductance Regulator (CFTR), a cyclic adenosine monophosphate–regulated ion channel, which primarily acts as a chloride channel and controls the movement of salt and water into and out of the epithelial cells of several glands. These mutations can cause abnormal functioning of the chloride channel and therefore an alteration of the sweat chloride ion secretion. Symptoms and signs that are commonly reported include persistent lung infections, pancreatic insufficiency, and high levels of sweat chloride [2]. However, the many different mutations causing the disease can also be responsible for mild or atypical symptoms [3–7], which can make the identification of this disease challenging. A conclusive diagnosis of CF should involve a combination of clinical presentation, genetics, and laboratory testing. The standard laboratory assays for CF consist in the measurement of chloride in sweat [8–11], which is often collected by the absorption method of Gibson and Cooke (proposed in 1959), based on the use of pilocarpine iontophoresis for sweat stimulation, that is generally regarded as the reference method for sweat testing [12]. More recently, a sweat stimulation and collection procedure (1982) employing the Wescor (Logan, UT, USA) Macroduct system has been introduced [13]. Despite the significant differences between these two methods that have an impact on both the sample preparation procedure and the chloride quantification method, a good agreement between them was observed [9]. CF is unlikely in individuals from all age groups with sweat chloride concentration levels < 30 mM. This is a considerable change with respect to previous guidelines which, for subjects over 6 months old, considered 40 mM as the cutoff value; this change was determined by the diagnosis of CF in patients with chloride values in the 30–39 mM range [14]. The current guidelines drawn up by the authoritative Clinical and Laboratory Standards Institute (CLSI) clearly identify the procedures for sweat collection [15], but no standardized methodology is proposed for chloride assay. However, among the common detection technologies for sweat chloride analysis, coulometry probably is the most widespread, but colorimetry and ion-selective electrode (ISE) are also considered acceptable. Actually, coulometry is the only method of sweat chloride analysis described in the CLSI guidelines [15], which also state “a concern when using ISEs to measure sweat chloride that the sensitivity at the lower concentrations could compromise the accuracy and precision of the results.” Also, colorimetry could provide satisfactory results, but accredited centers using such a technique usually are not involved in cystic fibrosis [16]. Due to the critical sensitivity of the analytical methods based on the abovementioned techniques, CLSI guidelines recommend the discarding of sweat samples weighing < 75 mg, often collected in infants [15]. In this frame, the high accuracy, sensitivity, linear dynamic range, and throughput of inductively coupled plasma mass spectrometry (ICP-MS) make this technology the best candidate to provide a top reference method to quantify chloride in sweat, allowing the quantification of chloride even in samples weighting < 75 mg. In general, ICP-MS is highly cost-effective and the operating costs of the clinical laboratory could greatly benefit from its high versatility, which allows performing different assays, usually carried out by several machines, by just one instrument. For instance, one of these assays could be the quantification of metal elements in biological fluids that are usually quantified individually by atomic absorption and that can be assessed simultaneously by ICP-MS multielemental analysis.

The use of ICP-MS for the sweat chloride assay is not a novelty if associated with the Wescor Macroduct sweat collection system [17, 18]. As an alternative, we propose here a validated ICP-MS-based method for the quantification on a routine basis of sweat chloride samples collected by the Gibson and Cooke technique.

## Materials and method

### Reagents and materials

NaCl, TraceCERT^®^-grade Chlorine Standard for IC in water, TraceCERT^®^-grade Gallium Standard for ICP, HNO_3_ (69% w/w), and NH_4_OH were from Sigma-Aldrich (St. Louis, MO, USA). Deionized water was produced by a DIA^®^ M deionizer from Quality Invents S.r.l. (Arluno, Italy), Argon ALPHAGAZ 1 grade (99.999% pure) was from Air Liquide (Milan, Italy), and Helium 5.6 purity grade (99.9996%) was from SOL (Monza, Italy). Three sweat quality controls with nominal concentrations of chlorine in the range 22.6–30.9 mM, 38.2–49.4 mM, and 78.8–102.4 mM were from LTA S.r.l (Milan, Italy); two ISE quality controls with nominal concentrations of 80 mM and 120 mM were from Roche Diagnostics GmbH (Basel, Switzerland). Whatman (Maidstone, UK) provided filter paper grade 40, 8 μm, used for sweat collection.

### Instrumentation

The chlorine quantifications based on the off-line internal standard technique were performed by an Agilent Technologies 7900 ICP-MS (Santa Clara, CA, USA), equipped with an ASX-500 Series autosampler and a peristaltic pump for sample injection. The analyses were preceded by an autotuning, which is an automated software routine that is able to optimize instrumental parameters to obtain the maximum level of sensitivity correlated with the maximum performance in terms of polyatomic interferences removal, and the acquisitions made use of the parameters reported in Table 1. System control and data acquisition and processing were carried out by the MassHunter 4.2 Workstation^®^ version C.01.02 software.

**Table 1:** ICP-MS operative parameters

Parameter	Value
Carrier gas flow rate	0.89 L/min (Ar)
Aerosol dilution flow rate	0.28 L/min (Ar)
Plasma gas flow rate	15 L/min (Ar)
collision gas flow rate	4.3E−3 L/min (He)
RF power	1600 W
Stabilization time	20 s
Peak pattern	3 points
Replicates	10
Integration time for ^35^Cl	1 s
Integration time for ^71^Ga	0.2 s
Sweeps per replicate	100
Peristaltic pump speed	0.1 rps

### Study population

Sweat samples from 50 healthy volunteers, aged 20–65 years (36.84 ± 12.34), and two individuals with suspected CF were analyzed. Patient 1 was a 24-year-old male with a history of chronic rhinitis and recurrent wheezing with wet cough from early childhood. Traditional sweat testing in the first year of life showed borderline values, i.e., 45 mM, 32 mM, and 36 mM when he was 1 month, 2 months, and 7 months old, respectively. When he was 10-years old, a computed tomography (CT) scan showed bronchiectasis in the middle and lower lobes and, more recently, genetic testing for CFTR mutations revealed a common heterozygous ΔF508 mutation (c.1521_1523delCTT, p.Phe508del). When he was 23 years old, a diagnosis of pansinusitis with nasal polyps was made by CT scan. Patient 2 was a 56-year-old woman with a history of chronic wet cough and recurrent fever, started when she was 55, after an acute respiratory infection. A CT scan performed 6 months after symptoms onset showed bronchiectasis in the middle lobe and multiple consolidations in the right upper lobe and lingula.

### Sample collection

Sweat samples were collected by the pilocarpine iontophoresis test (QPIT) [12]. Briefly, after cleaning the skin of the forearm by deionized water and drying it by a gauze, silver electrodes were placed, covered by a gauze soaked in 0.5% pilocarpine nitrate solution. Iontophoresis was performed by supplying a 4 mA current for 5 min After cleaning again the skin with deionized water, a pre-weighed filter paper sheet (4 × 3 cm, 95 g/m^2^) was placed onto the iontophoresed skin area, covered with Parafilm M (Bemis Company, Inc., Neenah, WI, USA), and kept in position for 30 min The moist filter paper was removed from the skin and secured in a pre-weighed 50-mL tube with a tightly fitting lid, and weighed again. Sweat samples weighing 75 to 300 mg were stored for further analysis.

### Preparation of calibrators and QCs

The NaCl stock solution was prepared at the concentration of 280.6 mM in deionized water. It was stable at least for 1 month when stored at room temperature in a polyethylene bottle. The IS stock solution containing Ga at the concentration of 28.7E−3 mM was prepared adding 4 μL of TraceCERT^®^ solution (1 mg/mL) to 1996 μL of deionized water. Such solution was stable for at least 1 month at room temperature, too. The calibration curve of Cl was built by six standard solutions, each of them prepared by parallel dilution with a deionized water solution of ammonium hydroxide, this latter having a final concentration of approximately 0.25% (w/w): 8.5E−3 mM (L1), 17.0E−3 mM (L2), 34.0E−3 mM (L3), 68.0E−3 mM (L4), 136.0E−3 mM (L5), 272.0E−3 mM (L6). Each calibrator contained IS at the concentration of 1.74E−5 mM. The calibrators were obtained according to a dilution procedure based on three consecutive steps. *Step 1* is as follows: 250 μL, 500 μL, 1000 μL, 2000 μL, 4000 μL, and 8000 μL of NaCl stock solution were all diluted with deionized water up to a final volume of 10 mL. *Step 2* is as follows: 200 μL of the solutions from *step 1*, 100 μL of the IS stock solution, and 3000 μL of deionized water were put into a 50-mL tube containing a 4 × 3 cm filter paper sheet, equivalent to that used for the sampling by Gibson and Cooke method, and the tube was then submitted to a 40-min stirring in a VDRL 711/D+ orbital stirrer (Asal, Cernusco sul Naviglio, Italy), at 150 rpm. Then, a filter paper was squeezed by disposable plastic forceps and removed from the tube. *Step 3* is as follows: 200 μL of the solutions from *step 2* were added with 9800 μL of a freshly prepared ammonium hydroxide 0.25% (w/w), thus obtaining the abovementioned calibrators. Six quality controls were prepared according to *steps 2* and *3* of the preparation procedure used for calibrators. Three of them were prepared at the operative concentration levels in the range 27.4E−3 to 37.5E−3 mM (QC1), 46.3E−3 to 59.9E−3 mM (QC2), and 95.5E−3 to 124.1E−3 mM (QC3), from the LTA sweat quality controls with nominal concentrations of chlorine in the range 22.6–30.9 mM (QC1′), 38.2–49.4 mM (QC2′), and 78.8–102.4 mM (QC3′), respectively. By the same procedure, two more quality controls at the operative concentration levels of 97.0E−3 mM (ISE1) and 145.5E−3 mM (ISE2) were prepared from the Roche commercial quality controls with nominal concentrations of 80 mM and 120 mM, respectively, and one more low-level quality control, CS, was prepared at the concentration of 34.2E−3 mM, from a certificated chlorine solution with a nominal concentration of 28.2 mM.

### Sample preparation

Sweat samples were prepared according to *steps 2* and *3* of the calibrators’ preparation procedure, except the amounts of IS stock solution and deionized water in *step 2*, which were proportional to the weight of collected sweat. In practice, considering a sweat density of 1 g/mL, a volume of IS stock solution and a volume of deionized water respectively corresponding to 50% and 1500% of the sweat volume were put into the 50-mL tube containing the moist filter (e.g., when 100 mg of sweat was collected, 50 μL of IS stock solution and 1500 μL of deionized water were necessary). A total of 1000 μL of the final solution was placed into the autosampler for the analysis.

### Chloride quantification

As described above, samples were prepared using the same preparation procedure of calibrators and quality controls, submitting all of them to the same dilution factor, whose total value was 825. Hence, although the calibration curve was built by calibrators L1 ÷ L6 (8.5 ÷ 272.0E−3 mM), the concentration values attributed to them in the quantitative section of the MassHunter 4.2 Workstation^®^ software were those of L1′ ÷ L6′ (7.0 ÷ 224.5 mM). This artifice avoided tedious calculations involving the sample dilution factors of both *steps 2* and *3* and allowed getting concentration values of chlorine immediately, in both samples and quality controls.

In case of Cl concentration greater than 40 mM, sweat crystallization test was performed as a confirmation test [19, 20]. Briefly, the test consisted in the observation by a polarized light optic microscope, of a droplet of sweat, placed on a slide and dried at room temperature. The test was considered negative when the obtained crystals showed a regular cuboid form and positive when exhibited a dendritic form or a fern pattern [20], with a variable score from 1 (+) through 4 (+) [21].

Genetic testing by next-generation sequencing was also carried out on positive patients.

### Method validation

To ensure data reliability, reproducibility, and robustness, a detailed validation of the analytical procedure was performed, with reference to selectivity, accuracy, precision, sensitivity, and stability, in compliance with EMA guidelines [22].

Selectivity is defined as the ability of analytical method to measure and differentiate the analyte and the IS from other components in the sample, behaving as potential interfering compounds, which affect the accuracy of the measurements, mainly when measuring low levels. It was tested by evaluating the method performances with the main stable isotopes for Cl, i.e., ^35^Cl and ^37^Cl, and with some stable isotopes suitable as an IS, such as ^69^Ga and ^71^Ga, ^45^Sc, ^72^Ge, and ^103^Rh [17].

Linearity of an analytical method is its capability to obtain results directly proportional to the concentrations of the analyte in the sample within a definite range. It was evaluated by monitoring ^35^Cl as an analyte and ^71^Ga as an IS, because they offered the best performances in terms of signal stability, signal-to-noise ratio, and precision of the measurements. The linearity of Cl, namely ^35^Cl, was tested within the calibration curve range, i.e., from 8.5E−3 mM through 272.0E−3 mM effective concentration.

Accuracy of an analytical method is the closeness of the experimental value to the nominal concentration of the analyte, expressed in percentage (accuracy %); precision represents the closeness among repeated individual measurements of the analyte and it is expressed as percent relative standard deviation (RSD%). Intra- and inter-day accuracy and precision were evaluated by five-replicate injection and three-replicate analyses in three different days, respectively, of QC1, QC2, QC3, CS, ISE1, and ISE2.

Limits of blank (LOB), of detection (LOD), and of quantitation (LOQ) of the analytical method were also achieved [23]. LOB is defined as the highest apparent analyte concentration expected to be found when replicates of a blank sample, containing no analyte, are tested. LOD is the lowest analyte concentration clearly distinguishable from LOB, and LOQ is the lowest concentration at which the analyte can be reliably quantified, which usually is ≥ LOD. LOB and LOD were calculated by using the following equations: LOB = mean _blank_ + 1.645(SD _blank_), LOD = LOB + 1.645(SD _low concentration sample_), where mean _blank_ and SD _blank_ were estimated by measuring 15 replicates of a blank sample and calculating the mean result and the standard deviation, respectively, and SD _low concentration sample_ was assessed by measuring 15 replicates of lowest calibrator [17, 23, 24]. LOQ was assessed at a concentration level above that of LOD and with a reliable and accurate signal. Its concentration value was then assigned to the lowest level (7.0 mM) calibrator.

To ensure an adequate sample stability, the weighed samples were stored for up to 72 h at refrigerator temperature (4 °C) prior to the analysis, as indicated in the CLSI guidelines [15]. However, experimental data in the literature demonstrated that chlorine can be measured within 5 days from the collection, without a significant affection on the reliability of results, irrespective of storage conditions [25]. Anyway, the stability of QC1, QC2, QC3, CS, ISE1, and ISE2 was evaluated by measuring the concentration of chlorine in freshly prepared solutions and after 1 and 7 days of storage at refrigerator temperature.

## Results

### The analytical method

The analytical method was designed to be simple and time- and cost-effective, to make it suitable for routine chloride analysis. The preparation of both calibrators and quality controls by the same procedure used for samples, as well as the use of an internal standard, resulted crucial to achieve satisfactory results in the quantification of the samples; the high analytical sensitivity of the method allows analyzing samples containing less than 75 mg of sweat, which are often collected in newborns and infants.

### Method validation

#### Selectivity

The main interferents in ICP-MS are ions having the same *m*/*z* of either the analyte or IS. According to the relative abundance of the natural isotopes, the monitored isotopes ^35^Cl, ^37^Cl, ^69^Ga ^71^Ga, ^45^Sc, ^72^Ge, and ^103^Rh do not suffer from significant singly charged monoatomic interferences. Several polyatomic interfering compounds might affect the detection; those more frequently described [26] consist in precursors from different sources (Table 2), such as the sample matrix, solvents, plasma gases, and atmospheric gases. Agilent 7900 ICP-MS is equipped with a collision cell that, when filled with a non-reactive gas, such as helium, exerts a kinetic energy discrimination (KED) and selectively attenuates the contributions of the polyatomic interferences [27]. Polyatomic ions are larger than both analyte and IS ions of the same mass and passing through the cell, they collide more frequently with inert gas molecules and emerge from the cell with a lower kinetic energy with respect to the ions of interest and can be selectively excluded from the ion beam by applying a suitable bias voltage at the cell exit. In contrast, the use of KED causes also significant analyte and IS sensitivity losses, which were not critical for our method. Also, doubly charged monoatomic ions could act as interferents (Table 3). Their cross section usually is comparable with that of the analytes, but their greater masses provide them with higher kinetic energies, which allows them to pass through the cell and reach the mass analyzer [28]. Thus, ^70^Zn^2+^ and ^70^Ge^2+^ could potentially interfere with ^35^Cl that comparative tests between ^35^Cl and ^37^Cl ions suggested to measure as an analyte, since it provided a better sensitivity, signal stability, and reproducibility with respect to ^37^Cl. However, the lowest second ionization potentials of Zn and Ge, respectively 17.96 eV and 15.93 eV, are higher than the first ionization potential of the plasma-forming gas Ar (15.76 eV), which is the limiting value of the second ionization potential. This makes the formation of ^70^Zn^2+^ and ^70^Ge^2+^ quite improbable [29]. The potential interferents of ^71^Ga, which was the most suitable IS, were ^142^Ce^2+^ and ^142^Nd^2+^, whose formation is justified by the lowest second ionization potentials of Ce and Nd, respectively 10.85 eV and 10.73 eV. Theoretically, the interference of these ions could be avoided by using the ICP-MS-MS technology [30], which, anyway, for our purpose is not strictly necessary, since the concentration of these two rare earth elements in the injected samples usually is insignificant and affects little the signal of the IS, as proved by the method accuracy.

**Table 2:** Monocharged polyatomic ions, which could interfere with the isotopes of interest in ICP-MS

Isotope	*m*/*z*	Interferences
^35^Cl	35	^16^O^18^O^1^H^+^, ^34^S^1^H^+^
^37^Cl	37	^36^Ar^1^H^+^, ^36^S^1^H^+^
^45^Sc	45	^12^C^16^O_2_^1^H^+^, ^28^Si^16^O^1^H^+^, ^29^Si^16^O^+^, ^14^N_2_^16^O^1^H^+^, ^13^C^16^O_2_^+^
^69^Ga	69	^35^Cl^16^O^18^O^+^, ^35^Cl^17^O_2_^+^, ^37^Cl^16^O_2_^+^, ^36^Ar^33^S^+^, ^33^S^18^O_2_^+^, ^34^S^17^O^18^O^+^, ^36^S^16^O^17^O^+^, ^33^S^36^S^+^
^71^Ga	71	^35^Cl^18^O2^+^, ^37^Cl^16^O^18^O^+^, ^37^Cl^17^O_2_^+^, ^36^Ar^35^Cl^+^, ^36^S^17^O^18^O^+^, ^38^Ar^33^S^+^
^72^Ge	72	^36^Ar_2_^+^, ^37^Cl^17^O^18^O^+^, ^35^Cl^37^Cl^+^, ^36^S^18^O_2_^+^, ^36^S_2_^+^, ^36^Ar^36^S^+^, ^56^Fe^16^O^+^, ^40^Ar^16^O_2_^+^, ^40^Ca^16^O_2_^+^, ^40^Ar^32^S^+^
^103^Rh	103	^40^Ar^63^Cu^+^

**Table 3:** Doubly charged monoatomic ions, which could interfere with the isotopes of interest in ICP-MS

Isotope	*m*/*z*	Interferences
^35^Cl	35	^70^Zn^2+^, ^70^Ge^2+^
^37^Cl	37	^74^Ge^2+^, ^74^Se^2+^
^45^Sc	45	^90^Zr^2+^
^69^Ga	69	^138^Ba^2+^, ^138^La^2+^, ^138^Ce^2+^
^71^Ga	71	^142^Ce^2+^, ^142^Nd^2+^
^72^Ge	72	^144^Nd^2+^, ^144^Sm^2+^
^103^Rh	103	^206^Pb^2+^

#### Linearity

The calibration curve used to quantify chloride in the samples showed a good linearity in the range 7.1–224.5 mM, corresponding to concentrations in the range 8.5E−3 to 272.0E−3 mM effectively injected into the ICP-MS instrument, with a reproducible slope and a correlation coefficient (R) always greater than 0.999. The linear regression with no weighing provided a curve equation *y* = *mx* + *q*, with *m* = 0.0187 ± 0.0014 and *q* = 0.0689 ± 0.0337.

#### Accuracy and precision

As shown in Table 4, the intra- and inter-day precision and accuracy were satisfactory. Precision exhibited a RSD always < 6%, while accuracy was in the range 99.7–103.8%. Accuracy was evaluated just for CS and ISE quality controls, as certificate of analysis of QC quality control provided concentration ranges instead of nominal concentration values.

**Table 4:** Accuracy and precision in the assessment of chlorine

	Nominal concentration (mM)	Mean concentration (mM)	RSD (%)	Accuracy (%)
Intra-day variation				
QC1	22.6–30.9	29.4	1.4	
QC2	38.2–49.4	48.4	0.5	
QC3	78.8–102.4	97.2	1.6	
CS	28.2	27.9	1.2	99.8
ISE1	80	80.7	4.2	103.7
ISE2	120	119.6	3.9	99.7
Inter-day variation				
QC1	22.6–30.9	30.8	1.5	
QC2	38.2–49.4	48.1	0.6	
QC3	78.8–102.4	95.4	1.4	
CS	28.2	28.6	3.9	102.1
ISE1	80	83.1	6.0	103.8
ISE2	120	120.1	4.2	100.1

#### LOB, LOD, and LOQ

Limit of blank, limit of detection, and limit of quantitation of the analytical method were 2.1 mM, 3.2 mM, and 7.0 mM, respectively. Interestingly, the real concentrations injected into the mass spectrometer were 825 folds lower, correspondent to 3.9E−3 mM for LOD and 8.5E−3 mM for LOQ.

#### Stability

No significant difference in the concentration of chlorine was observed by comparing QC1, QC2, QC3, CS, ISE1, and ISE2 to themselves with different times of storage (freshly prepared, day 1, day 7), suggesting that quality controls were stable for at least up to 7 days at refrigerator temperature.

### Quantification of clinical samples

The mean chloride concentration among the 50 healthy volunteers was 15.7 ± 7.4 mM, (25–75th percentile 10.1–19.3 mM, range 2.8–37.4 mM); in contrast, two patients with suspected cystic fibrosis had respectively a sweat chlorine value of 65.6 mM (patient 1) and 81.2 mM (patient 2). These results were further confirmed by crystallization test, with a score of 4 (+) being assigned to both patients.

For patient 1, further genetic testing by next-generation sequencing on CFTR gene showed a D1152H heterozygous mutation (c.3454G>C, p.Asp1152His) [31, 32].

### Cross-validation

**Fig. 1:**
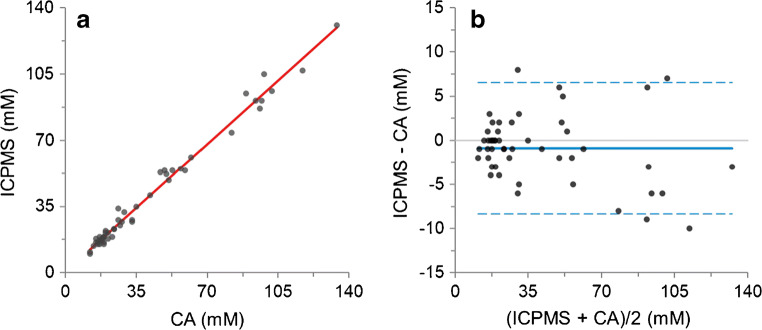
Comparison between ICP-MS and Chloride Analyser (CA) in the assay of chloride: linear regression analysis provides a regression line with a slope of 0.9626 (0.9297 to 0.9954), an intercept of 0.6548 (− 1.044 to 2.354), and a correlation coefficient (R) of 0.993 (**a**), while Bland-Altman plot exhibits a bias of − 0.9 units and 95% limits of agreement of − 8.3 and 6.6 (**b**)

In order to cross validate the method [22], 50 serum samples from 30- to 50-day-old newborns with chloride concentration values in the range 10 ÷ 131 mM, were assayed with either our ICP-MS method or the Chloride Analyser (Model 926S Mk II Chloride Analysers, Sherwood Scientific Ltd., Cambridge, UK), the latter method usually used for cystic fibrosis newborn screening in Tuscany (Italy), and their results were compared with each other. The normality of data distribution was assessed using Kolmogorov-Smirnov and Shapiro-Wilk tests. Mean chloride concentration measured with ICP-MS and Chloride Analyser (CA) were 40.12 ± 30.89 (median 26, range 10–131) mM and 41.00 ± 31.87 (median 26, range 12–134) mM, respectively. The difference was not statistically significant (*P* = 0.096, Wilcoxon’s signed-rank test). The intra-class correlation coefficient (ICC) for chloride concentration was 0.996 (95% confidence interval [CI] = 0.993 ÷ 0.998, *P* < 0.0001). The linear regression line represents the relationship between ICP-MS and Chloride Analyser (CA), shown in Fig. 1a, exhibited an equation with a slope of 0.9626 (95% confidence interval [CI] = 0.9297 ÷ 0.9954, *P* < 0.0001), an intercept of 0.6548 (95% confidence interval [CI] = − 1.044 ÷ 2.354, *P* = 0.4423), and a correlation coefficient (*R*) of 0.993. The Bland-Altman plot, reported in Fig. 1b, confirmed the analogous concentration levels for CA and ICP-MS, since bias had a value of − 0.9 (95% confidence interval [CI] = − 1.96 ÷ 0.20), with lower and upper limit of agreement of − 8.3 (95% confidence interval [CI] = − 10.18 ÷ − 6.47) and 6.6 (95% confidence interval [CI] = 4.71 ÷ 8.42), respectively [33]. Results are fully in compliance with EMA guidelines [22], which, for study samples, require a difference between the two values obtained by the different assays within 20% of the mean for at least 67% of the repeats, since 96% of the obtained pairs of values were within 20% of the mean.

## Conclusions

Inductively coupled plasma mass spectrometry (ICP-MS) is increasingly used in the clinical laboratory, mainly for assessments on a routine basis of oligoelements and isotopes of biomedical interest [34–36]. Its versatility, as well as its sensitivity, selectivity, and accuracy, can be advantageously utilized also for sweat chloride assay, providing a significant advancement over the commonly used ion-selective procedures, whose analytical performances at low concentrations can be quite critical in terms of quantification accuracy. This is the case of coulometry-based assay (namely Chloride Analysers, used in the newborn screening laboratory of Tuscany) that was chosen as the reference method to cross validate our ICP-MS-based method. The results, which in compliance with the CLSI guidelines were obtained with sweat samples ≥ 75 mg, confirmed the suitability of the ICP-MS method as a diagnostic tool. The good correlation between the two techniques confirms that the recommended reference values are suitable also for the ICP-MS assay, which was adopted for the clinical routine of the University Hospital of Pisa. Its improved analytical performances above the approved quantitative methods for Cl, which derive from serum-based methods whose Cl concentration curve is extrapolated to fit the lower Cl concentrations detected in sweat [16], will probably allow a better definition of the reference values of sweat chloride, especially in adolescents and adults. The increased analytical sensitivity, and the possibility to improve method sensitivity even further by reducing the sample dilution, could allow a reliable quantification of samples < 75 mg, potentially influencing the future guidelines concerning the collection methods. Moreover, the accurate quantification by ICP-MS as the sweat chloride concentration may reveal useful to improve the prediction of survival of patients with unclassified CFTR genotype [37]. As far as the price of a single determination is concerned, considering the workload of our instrumentation and personnel, we estimated it around 9 euro that at best of our knowledge is equivalent, if not lower, to those of the routine diagnosis of cystic fibrosis. We must emphasize that the attainment of reference results by ICP-MS is subjected to the availability of highly trained personnel, with a good knowledge of the technique and able to solve the frequent problems that inevitably occur with these instruments.

## References

[1] ECFSPR Annual Report 2015, Zolin A, Orenti A, Naehrlich L, van Rens J et al. 2017.

[2] Rosenstein BJ, Cutting GR (1998). The diagnosis of cystic fibrosis: a consensus statement. Cystic Fibrosis Foundation Consensus Panel. J Pediatr.

[3] Ratjen F, Döring G (2003). Cystic fibrosis. Lancet..

[4] De Boeck K, Wilschanski M, Castellani C, Taylor C, Cuppens H, Dodge J, Sinaasappel M (2006). Cystic fibrosis: terminology and diagnostic algorithms. Thorax..

[5] Grosse SD, Boyle CA, Botkin JR (2004). Newborn screening for cystic fibrosis: evaluation of benefits and risks and recommendations for state newborn screening programs. MMWR Recomm Rep.

[6] Crossley JR, Smith PA, Edgar BW, Gluckman PD, Elliott RB (1981). Neonatal screening for cystic fibrosis, using immunoreactive trypsin assay in dried blood spots. Clin Chim Acta.

[7] Therrell BL, Hannon WH, Hoffman G, Ojodu J, Farrell PM (2012). Immunoreactive trypsinogen (IRT) as a biomarker for cystic fibrosis: challenges in newborn dried blood spot screening. Mol Genet Metab.

[8] Farrell PM, Rosenstein BJ, White TB, Accurso FJ, Castellani C, Cutting GR, Durie PR, Legrys VA, Massie J, Parad RB, Rock MJ, Campbell PW (2008). Guidelines for diagnosis of cystic fibrosis in newborns through older adults: Cystic Fibrosis Foundation consensus report. J Pediatr.

[9] Nguyen-Khoa T, Borgard JP, Marchand M, Sitruk-Khalfon D, Feuillet MN, Feldmann D, Vassault A, Rota M (2012). Analytical quality of assays and comparison of procedures for the sweat test. Ann Biol Clin (Paris).

[10] Mattar AC, Leone C, Rodrigues JC, Adde FV (2014). Sweat conductivity: an accurate diagnostic test for cystic fibrosis?. J Cyst Fibros.

[11] Doorn J, Storteboom TT, Mulder AM, de Jong WH, Rottier BL, Kema IP (2015). Ion chromatography for the precise analysis of chloride and sodium in sweat for the diagnosis of cystic fibrosis. Ann Clin Biochem.

[12] Gibson LE, Cooke RE (1959). A test for concentration of electrolytes in sweat in cystic fibrosis of the pancreas utilizing pilocarpine by iontophoresis. Pediatrics..

[13] Barnes GL, Vaelioja L, McShane S (1988). Sweat testing by capillary collection and osmometry: suitability of the Wescor Macroduct system for screening suspected cystic fibrosis patients. Aust Paediatr J.

[14] Farrell PM, White TB, Ren CL, Hempstead SE, Accurso F, Derichs N, Howenstine M, McColley SA, Rock M, Rosenfeld M, Sermet-Gaudelus I, Southern KW, Marshall BC, Sosnay PR (2017). Diagnosis of cystic fibrosis: consensus guidelines from the Cystic Fibrosis Foundation. J Pediatr.

[15] CLSI. Sweat testing: sample collection and quantitative chloride analysis; approved guideline—third edition*.* CLSI document C34-A3. Clinical and Laboratory Standards Institute; 2009.

[16] Collie JTB, Massie RJ, Jones OAH, LeGrys VA, Greaves RF (2014). Sixty-five years since the New York heat wave: advances in sweat testing for cystic fibrosis. Pediatr Pulmonol.

[17] Collie JT, Massie RJ, Jones OA, Morrison PD, Greaves RF (2016). A candidate reference method using ICP-MS for sweat chloride quantification. Clin Chem Lab Med.

[18] Pullen NJ, Thurston V, Barber S (2013). Evaluation of an inductively coupled plasma mass spectrometry method for the analysis of sweat chloride and sodium for use in the diagnosis of cystic fibrosis. Ann Clin Biochem.

[19] Ferrer-Calvete J, Ribes C, Montero C (1990). The form of crystallization of perspiration in pancreatic cystic fibrosis. An Esp Pediatr.

[20] Ferrer-Calvete J, Ribes C, Montero C (1990). The sweat crystallization test in the diagnosis of cystic fibrosis. J Pediatr Gastroenterol Nutr.

[21] Kopito L, Ploss RS, Shwachman H (1967). Crystal forms in sweat from patients with cystic fibrosis. Bibl Paediatr.

[22] Guideline on bioanalytical method validation. Eur Med Agency 2015;4–10.

[23] Armbruster DA, Pry T (2008). Limit of blank, limit of detection and limit of quantitation. Clin Biochem Rev.

[24] Shrivastava A, Bupte VB (2011). Methods for the determination of limit of detection and limit of quantitation of the analytical methods. Chron Young Sci.

[25] Bergeron J, Bachmann LM, Miller WG (2011). Influence of sample storage conditions on sweat chloride results. Clin Chem.

[26] May TW, Wiedmeyer RH (1998). A table of polyatomic interferences in ICP-MS. At Spectrosc.

[27] Yamada N (2015). Kinetic energy discrimination in collision/reaction cell ICP-MS: theoretical review of principles and limitations. Spectrochim Acta Part B At Spectrosc.

[28] Blokhin MG, Zarubina NV, Mikhailyk PE (2014). Inductively coupled plasma mass spectrometric measurement of gallium in ferromanganese crusts from the Sea of Japan. J Anal Chem.

[29] Pupyshev AA, Semenova EV (2001). Formation of doubly charged atomic ions in the inductively coupled plasma. Spectrochim Acta Part B At Spectrosc.

[30] Balcaen L, Bolea-Fernandez E, Resano M, Vanhaecke F (2015). Inductively coupled plasma - tandem mass spectrometry (ICP-MS/MS): a powerful and universal tool for the interference-free determination of (ultra)trace elements – a tutorial review. Anal Chim Acta.

[31] Lucarelli M, Porcaro L, Biffignandi A, Costantino L, Giannone V, Alberti L, Bruno SM, Corbetta C, Torresani E, Colombo C, Seia M (2017). A new targeted CFTR mutation panel based on next-generation sequencing technology. J Mol Diagn.

[32] Terlizzi V, Carnovale V, Castaldo G, Castellani C, Cirilli N, Colombo C, Corti F, Cresta F, D'Adda A, Lucarelli M, Lucidi V, Macchiaroli A, Madarena E, Padoan R, Quattrucci S, Salvatore D, Zarrilli F, Raia V (2015). Clinical expression of patients with the D1152H CFTR mutation. J Cyst Fibros.

[33] Giavarina D (2015). Understanding Bland Altman analysis. Biochem Med.

[34] Yu LL, Davis WC, Nuevo Ordonez Y, Long SE (2013). Fast and accurate determination of K, Ca, and Mg in human serum by sector field ICP-MS. Anal Bioanal Chem.

[35] Hastuti AAMB, Costas-Rodríguez M, Anoshkina Y, Parnall T, Madura JA, Vanhaecke F (2020). High-precision isotopic analysis of serum and whole blood Cu, Fe and Zn to assess possible homeostasis alterations due to bariatric surgery. Anal Bioanal Chem.

[36] Rodushkin I, Engström E, Baxter DC (2013). Isotopic analyses by ICP-MS in clinical samples. Anal Bioanal Chem.

[37] McKone EF, Velentgas P, Swenson AJ, Goss CH (2015). Association of sweat chloride concentration at time of diagnosis and CFTR genotype with mortality and cystic fibrosis phenotype. J Cyst Fibros.

